# Effect of a client-accessible record on autonomy of parents and adolescents in Dutch care for youth

**DOI:** 10.1093/eurpub/ckac129.647

**Published:** 2022-10-25

**Authors:** J Benjamins, A Haveman-Nies, E de Vet

**Affiliations:** 1 Icare JGZ, Meppel, Netherlands; 2 CHL, Wageningen University and Research, Wageningen, Netherlands; 3 GGD NOG, Warnsveld, Netherlands

## Abstract

**Background:**

In the Netherlands, since 2015, a paradigm shift towards client-centred ‘care for youth’ initiated a focus on client autonomy, enabling clients to make informed decisions in their care process. Client autonomy is assumed to positively correlate with mental and physical health and can be strengthened by autonomy-supportive behaviour from professionals. Aiming for client autonomy, three organizations developed an electronic health record (EPR-Youth), accessible for parents and adolescents. Although research shows that patient-accessible records contribute to patient autonomy, limited research is available about use of such records among adolescents. We investigated whether use of EPR-Youth in ‘care for youth’ contributed to experienced autonomy among adolescents and parents, and what role professional autonomy-supportive behaviour played.

**Methods:**

A mixed methods design combined baseline and follow-up questionnaires with focus group interviews, over a two-year period. Clients completed a questionnaire about experienced autonomy and portal use at baseline (1202 parents, 202 adolescents) and after one year (914 parents, 89 adolescents). Professionals completed questionnaires about autonomy-supportive behaviour at baseline (N = 100), after 5 months (N = 57) and 24 months (N = 110). After 14 months, focus group interviews were held with a purposive sample of parents (N = 8), adolescents (N = 4) and professionals (N = 12).

**Results:**

Twelve months after introducing EPR-Youth, higher autonomy scores were found among parents (Δ = 0·23; 95%CI=0·18-0·28; p < 0·001) and adolescents (Δ = 0·53; 95%CI=0·34-0·73; p < 0·001). Portal users showed higher scores than non-users. Focus group members reported a positive effect of portal use on client autonomy, which was strengthened by professional autonomy-supportive behaviour.

**Conclusions:**

The use of EPR-Youth was associated with increased autonomy among parents and adolescents. Autonomy-supportive professional behaviour enhanced this effect.

**Key messages:**

• The possible contribution of EPR-Youth to client autonomy is promising, but follow-up research is needed to strengthen evidence.

• Implementation of client-accessible records with the aim to enhance client/patient autonomy needs to address autonomy-supportive professional behaviour to increase impact.

